# MiR-30a-3p Negatively Regulates BAFF Synthesis in Systemic Sclerosis and Rheumatoid Arthritis Fibroblasts

**DOI:** 10.1371/journal.pone.0111266

**Published:** 2014-10-31

**Authors:** Ghada Alsaleh, Antoine François, Lucas Philippe, Ya-Zhuo Gong, Seiamak Bahram, Semih Cetin, Sébastien Pfeffer, Jacques-Eric Gottenberg, Dominique Wachsmann, Philippe Georgel, Jean Sibilia

**Affiliations:** 1 Immunorhumatologie moléculaire, INSERM UMR S_1109, Centre de Recherche en Immunologie et Hématologie, Faculté de Médecine, Fédération de Médecine Translationnelle de Strasbourg (FMTS), Université de Strasbourg, Strasbourg, France and Service de Rhumatologie, Centre National de Référence pour les Maladies Systémiques Autoimmunes Rares, Hôpitaux Universitaires de Strasbourg, Strasbourg, France; 2 Université de Strasbourg, UPR 9002 du CNRS Architecture et Réactivité de l'ARN, Institut de Biologie Moléculaire et Cellulaire, Strasbourg, France; University of Texas Health Science Center at Houston, United States of America

## Abstract

We evaluated micro (mi) RNA-mediated regulation of BAFF expression in fibroblasts using two concomitant models: (i) synovial fibroblasts (FLS) isolated from healthy controls (N) or Rheumatoid Arthritis (RA) patients; (ii) human dermal fibroblasts (HDF) isolated from healthy controls (N) or Systemic Sclerosis (SSc) patients. Using RT-qPCR and ELISA, we first showed that SScHDF synthesized and released BAFF in response to Poly(I:C) or IFN-γ treatment, as previously observed in RAFLS, whereas NHDF released BAFF preferentially in response to IFN-γ. Next, we demonstrated that miR-30a-3p expression was down regulated in RAFLS and SScHDF stimulated with Poly(I:C) or IFN-γ. Moreover, we demonstrated that transfecting miR-30a-3p mimic in Poly(I:C)- and IFN-γ-activated RAFLS and SScHDF showed a strong decrease on BAFF synthesis and release and thus B cells survival in our model. Interestingly, FLS and HDF isolated from healthy subjects express higher levels of miR-30a-3p and lower levels of BAFF than RAFLS and SScHDF. Transfection of miR-30a-3p antisense in Poly(I:C)- and IFN-γ-activated NFLS and NHDF upregulated BAFF secretion, confirming that this microRNA is a basal repressors of BAFF expression in cells from healthy donors. Our data suggest a critical role of miR-30a-3p in the regulation of BAFF expression, which could have a major impact in the regulation of the autoimmune responses occurring in RA and SSc.

## Introduction

TNFSF13B (also known as B cell-activating factor -BAFF, which will be the only nomenclature used thereafter in the manuscript) is a member of the TNF superfamily which plays a central role in the survival and homeostasis of transitional and naive B cells, plasmablasts and plasma cells [1]. BAFF is produced as a membrane-bound form or secreted by cells of hematopoietic origin, essentially monocytes, dendritic cells, macrophages and stimulated neutrophils [2], [3]. However non-immune cells, such as astrocytes, can also constitutively produce BAFF [4].

The finding that BAFF transgenic mice develop autoimmune manifestations exhibiting similarities with systemic lupus erythematosus (SLE) and Sjögren's syndrom (SS) suggested a critical role for this cytokine in autoimmune diseases [5]–[7]. Consistent with this observation, auto-reactive B cells have a greater dependency for BAFF compared to naive mature B cells and elevated levels of BAFF were detected in the serum of patients with SLE, rheumatoid arthritis (RA) and SS [8]. In addition, these increased BAFF levels correlated with high auto-antibody titers and disease activity [9]. In patients with systemic sclerosis (SSc), increased levels of BAFF were associated with worsening of the skin sclerosis [10], [11]. Similar results were obtained in experimental arthritis where overproduction of BAFF by dendritic cells and macrophages was demonstrated to play a crucial role in the disease [12]. Fibroblast-like synoviocytes isolated from RA patients (RAFLS) are characterized by their aggressive phenotype in response to various stimuli. In these inflammatory conditions, these cells also produce large amounts of cytokines including BAFF, thus enabling them to collaborate with autoimmune B cells [13]. Altogether, these data illustrate the central role played by BAFF in the pathogenesis of autoimmune diseases, which prompted the development of biological agents targeting BAFF. Belimumab, a humanized IgG1 monoclonal antibody which inhibits both soluble and membrane BAFF binding to BAFFR and TACI, was approved for the treatment of SLE [14]–[16].

However, modulation of BAFF activity for therapeutic purposes by targeting its synthesis has not yet been considered. The control of cytokine expression can occur at various levels including post-transcriptional regulation, which is now the focus of intense attention. The modulation of gene expression can take place at several levels, among which regulation by microRNAs (miRNAs) has gained increased interest in the recent years. MiRNAs are an evolutionarily conserved class of endogenous small non-coding RNAs. They are produced from long precursor molecules by the consecutive action of the RNase III enzymes DROSHA and DICER, before being loaded on an ARGONAUTE protein within the RNA-induced-silencing complex (RISC). The mature miRNA acts a guide for RISC to mediate destabilization and/or translational repression of target mRNAs. The regulation of miRNA expression is itself controlled at various levels such as transcription, processing or stability and can be influenced by various stress factors including inflammation. In addition, emerging data have identified an important contribution of miRNA to the development and control of the inflammatory response which position these small non-coding RNAs at the heart of feedback and feed-forward loops controlling the inflammation process in both immune and non immune cells. Recently, microRNAs (miRNAs) have emerged as a new class of cytokines regulators, although computational analysis indicates that the 3′UTR (Three prime untranslated region) of many cytokines lacks miRNA target sites. Indeed, miRNAs could also regulate cytokine expression by targeting ARE-binding proteins (ARE machinery components) such as TTP, AUF1 and members of the HuR family [17], [18].

In this study, we found that miR-30a-3p (and miR30d-3p and e-3p, which exhibit high sequence homology) is predicted to bind the 3′UTR region of BAFF transcripts. In SSc patients, we observed concomitant up-regulation of BAFF transcripts and decreased expression of miR-30a-3p in skin fibroblasts (HDF) isolated from SSc patients after cell stimulation with Poly (I:C) or IFN-γ. Analysis of synovial fibroblasts from RA patients yielded similar results. The prediction provided by bioinformatic tools and this inverse correlation between miR-30 family members and BAFF expression prompted us to analyze their likely direct interaction. To this end, we used reporter constructs containing a luciferase gene fused to the wild type or mutated full length human BAFF 3′UTR. Co-transfection of these reporter plasmids with miRNA mimic in cultured cells confirmed direct interaction. Furthermore, transfection of miR-30a-3p mimic or inhibitor in fibroblasts isolated from the skin or the synovium of healthy donors or from patients confirmed that this miRNA modulate IFN-γ- and Poly (I:C)-dependent BAFF expression. Finally, we report that miRNA-modulated BAFF secretion by activated skin or synovial fibrocytes significantly reduces the capacity of these cells to promote B cells survival, which provides physiological significance to our findings. Altogether, our data describe a novel mechanism involved in the regulation of BAFF expression and potentially provide an additional level of intervention for future therapeutic purposes.

## Materials and Methods

### Reagents

Cell culture media (RPMI 1640, M199 and DMEM), fetal calf serum (FCS), L-glutamine, penicillin, streptomycin, amphotericin B, TRIzol reagent and DiOC_6_ (3,3′-Dihexyloxacarbocyanine Iodide) were from Invitrogen (Cergy-Pontoise, France). LPS from *Salmonella abortus equi* and Propidium Iodide (PI) solution was obtained from Sigma Aldrich (Saint-Quentin-Fallavier, France). Synthetic bacterial lipopeptide Pam_3_CSK_4_ (BLP) was obtained from EMC Microcollections GmbH (Tuebingen, Germany). Polyinosine-Polycytidylic acid (Poly(I:C)) was obtained from InvivoGen (Toulouse, France). iScript cDNA Synthesis Kit and SsoAdvanced SYBR Green Supermix from Bio-Rad (Marnes-la-Coquette, France). The miScript System, miRNA mimc and Allstars negative control siRNA were obtained from Qiagen (Courtabeuf, France). miR-30a-3p antagonists were from Fisher scientific (Illkirch Cedex, France). Human Dermal Fibroblast Nucleofector kit was from Lonza (Cologne, Germany). The enzyme immunoassay kits for human BAFF, APRIL and IL-6 detection and recombinant IFN-γ were from R&D systems (Lille, France).

### Cell culture

Human FLS were isolated from synovial tissues from five different RA patients and from five healthy subjects at the time of knee joint arthroscopic synovectomy. RA patients were 3 female and two male, the average age is 49 years, and had all FR positive but only three were antiCCP positive. Human dermal fibroblasts (HDF) were obtained by biopsy of 4 mm diameter from the affected areas (dorsal forearm) of four patients with SSc (SScHDF)and from the corresponding area of three healthy subjects (NHDF). All SSc patients were female (average age52 years) and had diffuse cutaneous systemic sclerosis and anti-Scl70-positive antibodies. The total modified Rodnan skin scores were 29, 8, 25 and 14, and that for the biopsy area were 2, 1, 2, 2.Blood B cells were isolated from 6 healthy donors. Institutional ethics committee of the Hopitaux Universitaires de Strasbourg specifically approved this study.Written informed consent was obtained by all the participants of this study (patients and healthy donors). The diagnosis of RA and SSc was conformed to the revised criteria of the American College of Rheumatology (ACR). FLS, HDF and HEK293 cultures were done as previously described [19]. Experiments were performed between the 3^rd^ and the 9^th^ passage. During that time, cultures were constituted of a homogeneous population of fibroblastic cells, negative for CD16 as determined by FACS analysis. HEK293 cells were purchased from the American type culture collection (ATCC) and maintained in DMEM supplemented with 10% heat-inactivated FBS, 2 mM of L-glutamine, 40 U/ml penicillin and 50 mg/ml streptomycin. Cell number and cell viability were checked by the MTT (3-(4,5 dimethylthiazol-2-yl)-2,5-diphenyltetrazolium bromide) assay. Blood mononuclear cells were isolated from healthy blood donors by Ficoll-Paque centrifugation as described in standard protocols. B cells were then selected by negative sorting using EasySep Human B Cell Enrichment Kit (Stemcell Technologies). The efficacy of B cell isolation was determined by FACS analysis using anti-CD19 antibodies. The yield of isolated B cells was composed of 99% CD19^+^/CD3^−^ B cells and 0.01% of CD19^−^/CD3^+^ T cells.

### Stimulation of cells for total RNA extraction

FLS (2.10^5^ cells) and HDF (2.10^5^ cells) were seeded in 24-well plates and stimulated with medium alone or medium containing LPS (1 µg/mL), BLP (1 µg/mL), Poly(I:C) (10 µg/mL) and IFN-γ (0.1, 1 or 5 ng/mL). After a 6 h, 48 h and 72 h incubation period, total RNA was extracted using TRIzol according to the manufacturer's instruction.

### Real-time quantitative PCR (RT-qPCR)

Total RNA was reverse transcribed using the iScript cDNA Synthesis Kit according to the manufacturer's instructions (BioRad). Real-time quantitative RT-qPCR was performed in a total volume of 20 µL using SsoAdvanced SYBR Green Supermix (BioRad) and gene-specific primers: BAFF: 5′-TGAAACACCAACTATACAAAAAG-3′ and 5′-TCAATTCATCCCCAAAGACAT-3′;

*April*: 5′- CTCTGCTGACCCAACAAACA-3′ and 5′- CTCCTTTTCCGGGATCTCTC-3′;

*Gapdh*: 5′-GGTGAAGGTCGGAGTCAACGGA-3′ and 5′-GAGGGATCTCGCTCCTGGAAGA-3′

After an initial denaturing at 96°C for 10 min, the temperatures used were 95°C for 10 s, 60°C for 15 s, 72°C for 25 s using a Rotor-Gene 6000 real-time PCR machine (Corbett Life Science). Amplification products were detected as an increased fluorescent signal of SYBR Green during the amplification cycles. Results were obtained using Rotor-Gene 6000 Series Software and evaluated using Excel (Microsoft). Melting-curve analysis was performed to assess the specificity of PCR products.

Real-time quantitative PCR analyses for miRNAs were performed using the miScript System and the primers (Qiagen). RNA concentrations were determined with a NanoDrop instrument (NanoDrop Technologies). 500 ng of RNA per sample was used for the assays. Reverse transcriptase reactions and RT-qPCR were performed according to the manufacturer's protocols. Expression of endogenous U6snRNA was used for normalization. Relative expression was calculated using the comparative threshold cycle (Ct) method and fold induction in cells activated by Poly(I:C) or IFN-γ was obtained by calculating 2^−ΔΔCt^.

### Transfections and luciferase assay

Transient transfection of FLS or HDF with miR-30a-3p mimic (20 pM/sample), miR-30a-3p antagonists or with the negative controls was performed using the Human Dermal Fibroblast Nucleofector kit from Lonza as previously described [20]. Transfection of HEK293 cells was performed using Lipofectamine 2000 (Invitrogen) as previously described [19].

### B cells viability assay

FLS or HDF were transfected with miR-30a-3p mimic, miR-30a-3p antagonists or with the negative controls as described above and stimulated with medium alone or medium with Poly(I:C) (10 µg/mL) or IFN-γ (5 ng/mL) for 72 h. Supernatants (800 µL) were harvested and used to culture B cells (5×10^5^) for 72 h. In some experiments, anti-human BAFF antibodies or control IgG (R&D Systems) were added (10 ng/mL) to the supernatant. Then, B cells were stained with 3,3-dihexyloxacarbocyanine iodide (DiOC_6_) to assess the mitochondrial transmembrane potential, and with Propidium Iodide (PI) to assess membrane permeability, as described [21]. Briefly, cell suspensions were incubated with 40 nmol/L DiOC_6_ and 1 µg/ml PI for 15 min at 37°C, washed with FACS buffer and then analyzed on FACSCalibur (BD Biosciences). A lymphocyte gate was set using forward-angle and side-angle light scatter characteristics of lymphocytes. The vital B cells were brightly positive when stained with DiOC_6_ and excluded PI.

### Statistical analysis

Student's t test (two-tailed unpaired) was used to compare two independent groups using GraphPad 5 software. A probability (p) value of <0.05 was considered significant. *p<0.05; **p<0.01; ***p<0.001.

## Results

### Increased BAFF secretion by SScHDF after stimulation with Poly(I:C) or IFN-γ

**Figure 1:**
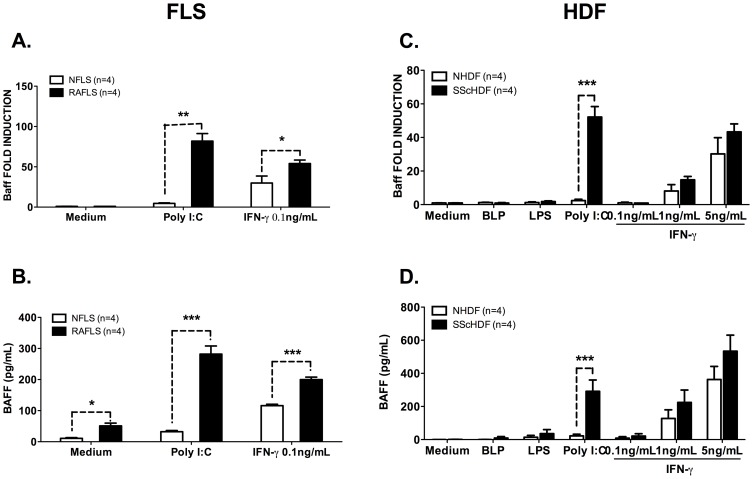
BAFF expression and secretion are up-regulated in Poly (I:C)- and IFN-γ-stimulated rheumatoid arthritis (RA) fibroblast-like synoviocytes (FLS) and systemic sclerosis (SSc) human dermal fibroblast (HDF). A, C. BAFF mRNA expression was determined by RT-qPCR in NFLS(n = 4) and RAFLS(n = 4) (A) or NHDF(n = 3) and SScHDF (n = 4) (C) stimulated (depending of the cell types) with BLP (1 µg/ml), LPS (1 µg/ml), Poly (I:C) (10 µg/mL) or IFN-γ (0.1, 1 or 5 ng/mL) for 72 h. Results were normalized to Gapdh and expressed as fold change compared with samples from cells incubated in medium. B, D. BAFF release was quantified by ELISA in culture supernatants of NFLS (n = 4) and RAFLS(n = 4) (B) or NHDF(n = 3) and SScHDF (n = 4) (D) in the same conditions as panels A and C. Data are expressed as the mean of triplicate samples ± SEM. *p<0.05, **p<0.01, ***p<0.001.

Upregulation of BAFF expression by Poly (I:C)- and IFN-γ-activated RAFLS, but not upon TLR2 or TLR4 activation, has been previously reported [13], [22]. To gain more insights into the physiopathological consequences of this observation, we first compared BAFF expression in FLS isolated from healthy donors or RA patients. As shown in figure 1A and B, we observed that IFN-γ-dependent BAFF expression reaches maximum levels in RAFLS, whereas FLS isolated from healthy donors (NFLS) exhibit reduced cytokine expression at both mRNA and protein levels. Of note, Poly (I:C) stimulation induced very limited BAFF expression and secretion by NFLS, while RAFLS appeared extremely responsive to this stimulus. Next, we tested whether this difference between a healthy and a pathological (inflammatory) state could also be observed in another fibroblastic cell type and for this, we chose Human Dermal Fibroblasts (HDF) isolated from skin biopsies harvested from healthy donors (NHDF) or from patients suffering from systemic sclerosis (SScHDF). IFN-γ stimulation (1 and 5 ng/mL) resulted in a comparable increased expression of BAFF transcripts (figure 1C) and cytokine secretion (figure 1D) by NHDF or SScHDF. Interestingly, up-regulation of BAFF transcripts and protein release in response to TLR3 triggering by Poly (I:C) was only detectable in SScHDF and not from healthy individuals. We also investigated here the ability of Bacterial LipoProteins (BLP, Pam_3_CSK4) or LipoPolysaccharide (LPS), which are respectively ligands for TLR2 and 4, to stimulate BAFF synthesis by NHDF and SScHDF. As seen in figure 1C and D, these PAMPs (Pathogen Associated Molecular Pattern) are not activators of BAFF transcription.

Thus, these results show that FLS and HDF from both healthy donors and RA or SSc patients can produce BAFF in response to IFN-γ stimulation, whereas BAFF transcription and protein secretion upon Poly (I:C) triggering occurred only in fibroblasts isolated from RA or SSc patients.

### miR-30a-3p is down-regulated in Poly(I:C)- and IFN-γ-activated RAFLS and SScHDF

To understand the mechanisms underlying Poly (I:C)- and IFN-γ-dependent BAFF induction, we then focused our work on miRNA-driven post-transcriptional regulation. A computer-assisted search for miRNAs predicted to target BAFF mRNA performed using microCosm (http://www.ebi.ac.uk/enright-srv/microcosm/htdocs/targets/v5) identified several miRNAs candidates: miR-144*, miR-452, miR-340, miR-202, miR-500, miR-626, miR-330-3p, miR-302c* and miR-30 family members (miR-30a, d and e which share the same seed sequence). To evaluate the possible involvement of these miRNAs in BAFF regulation, we first performed RT-qPCR analysis to quantify their expression in RAFLS and SScHDF treated with Poly(I:C) or IFN-γ for 6 h, 48 h and 72 h. This analysis revealed that miR-144*, miR-30d-3p, miR-340, miR-626, miR-330-3p and miR-302c* could not be detected in RAFLS and SScHDF (figure 1S). miR-202 and miR-500 were expressed constitutively but their expression did not change after activation by Poly(I:C) or IFN-γ in both cell type. Finally, miR-452 was upregulated in Poly (I:C) treated RAFLS.

**Figure 2:**
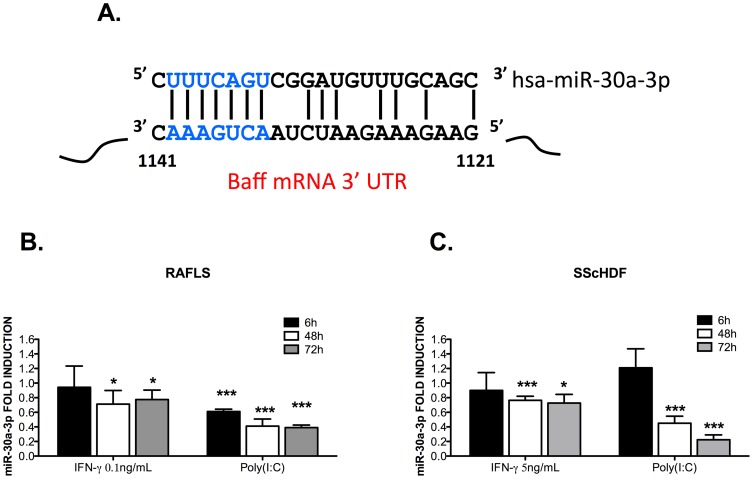
miR-30a-3p expression is down-regulated in Poly (I:C)- and IFN-γ-stimulated RAFLS and SScHDF. A. miR-30a-3p is predicted to target BAFF 3′ UTR mRNA. B, C. miR-30a-3p expression was determined by RT-qPCR in RAFLS (n = 4) (B) and SScHDF (n = 4) (C) stimulated with Poly (I:C) (10 µg/mL) or IFN-γ (0.1 or 5 ng/mL) for 6 h, 48 h and 72 h. Results were normalized to U6snRNA and expressed as fold change compared with samples from cells incubated in medium. Data are expressed as the mean of triplicate samples ± SEM. *p<0.05, ***p<0.001.

Interestingly, we noted that both miR-30a-3p and miR-30e-3p (data not shown), were significantly down-regulated in Poly (I:C)- and IFN-γ-activated, RAFLS (Figure 2B) and SScHDF (figure 2C) 48 h and 72 h after stimulation. These data, together with those illustrated in figure 1, indicate that BAFF transcripts and miR-30a-3p exhibit opposite expression patterns, therefore suggesting potential interactions. Given the strong similarities between miR-30a-3p and miR-30e-3p, we decided to focus on miR-30a-3p.

### miR-30a-3p directly interacts with the 3′UTR of BAFF mRNA

**Figure 3:**
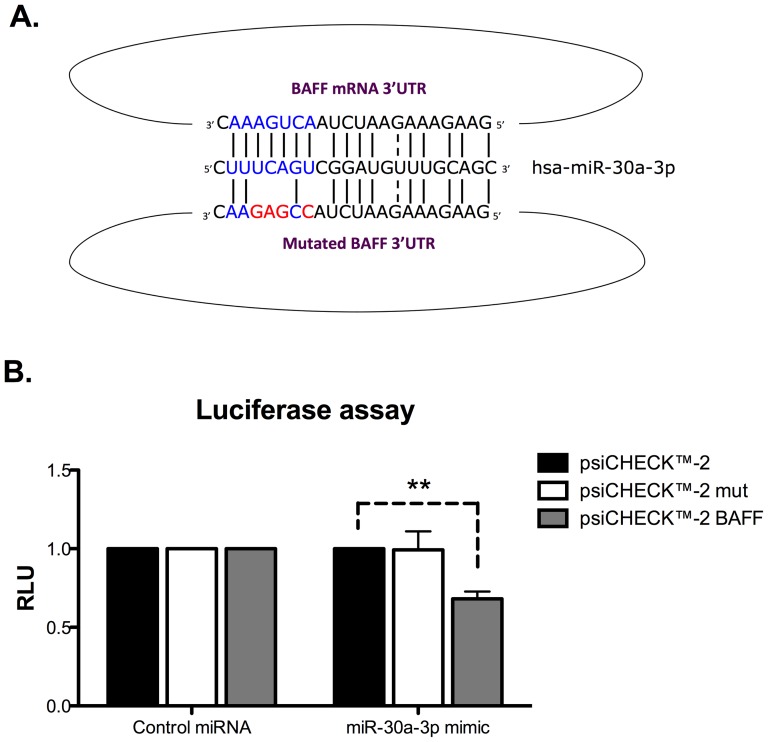
miR-30a-3p directly targets the 3′UTR of BAFF mRNA. A. Luciferase reporter constructs with wild-type or mutated (for miR-30-3p binding sites) BAFF 3′UTR were generated. B. HEK293 cells were transiently co-transfected with reporter constructs and with miR-30a-3p mimic (20 pM). Firefly Luciferase activities were measured 48 h after transfection and normalized to Renilla Luciferase expressed by the control psi-CHECK-2 vector devoid of 3′UTR sequences. Data are expressed as the mean of triplicate samples ± SEM and are representative of three independent experiments. **p<0.01.

To validate the involvement of miR-30a-3p in the regulation of *BAFF* expression, we generated luciferase reporter constructs (derived from the pSI-CHECK2 vector) containing the firefly luciferase gene fused to the entire human BAFF 3′UTR sequence and the renilla luciferase for normalization. We also generated a reporter construct in which a mutated version, designed to disrupt the predicted seed-match for miR-30a-3p of the human BAFF 3′UTR, was inserted (figure 3A). These plasmids were co-transfected in HEK293 cells with miR-30a-3p mimic or AllStars negative control siRNA (CT). In the presence of miR-30a-3p mimic, we observed a significant down regulation of the BAFF 3′UTR-controlled luciferase sensor, whereas luciferase expression upon transfection of the mutated form of the reporter vector remained unchanged (figure 3B). Altogether, these data suggest that BAFF transcripts can be directly targeted by miR-30a-3p for post-transcriptional regulation.

### miR-30a-3p modulates BAFF expression in RAFLS and SScHDF

**Figure 4:**
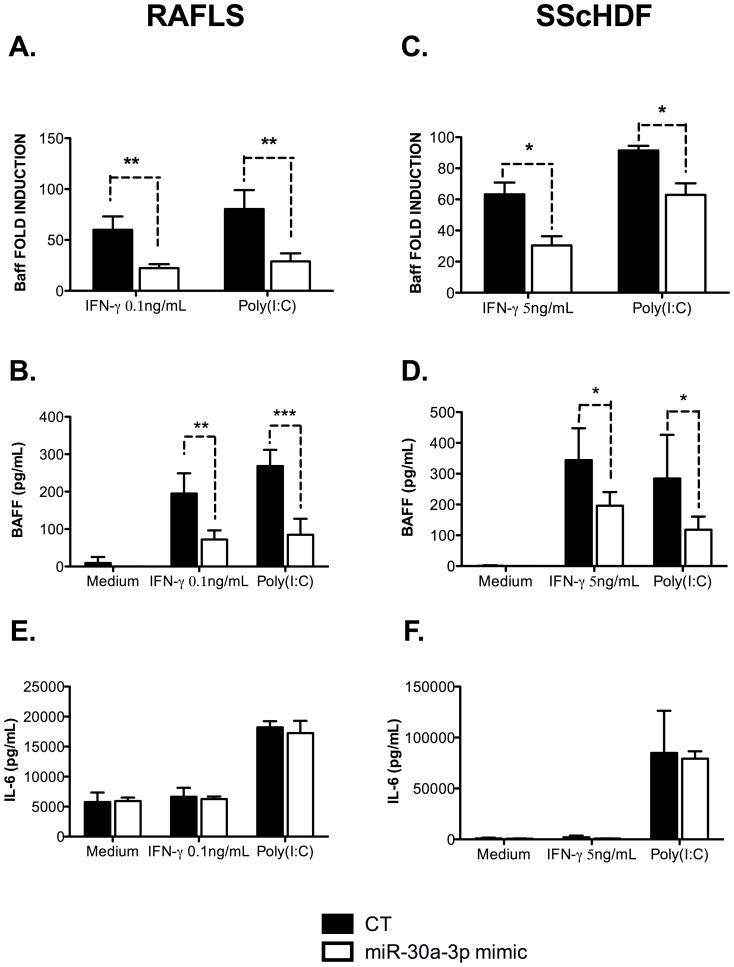
miR-30a-3p transfection affects BAFF mRNA expression and BAFF secretion in RAFLS and SScHDF. A, C. RAFLS (n = 5) (A) and SScHDF (n = 4) (C) were transfected with miR-30a-3p mimic (20 pM/sample) or with an AllStars negative control (CT). After 24 h, cells were activated with Poly (I:C) (10 µg/mL), IFN-γ (0.1 or 5 ng/mL, depending on cell types) or medium for 72 h. BAFF mRNA expression was determined by RT-qPCR. Results were normalized to Gapdh and expressed as fold change compared with samples from cells incubated with medium. B, D. BAFF release was determined by ELISA in culture supernatants in the same conditions as panel A and C. E, F. IL-6 release was determined by ELISA in culture supernatants in the same conditions as panel A and C. Data are expressed as the mean of triplicate samples ± SEM. *p<0.05; **p<0.01; ***p<0.001.

To assess the effect of miR-30a-3p on BAFF expression, we measured the production of BAFF mRNA by RT-qPCR in RAFLS and SScHDF transfected with miR-30a-3p mimic or with the AllStars negative siRNA control (CT). 24 h after transfection, cells were stimulated with Poly (I:C) or IFN-γ for 72 h. As seen in Figure 4, we found that overexpression of miR-30a-3p led to a global decrease in BAFF mRNA production and protein secretion by Poly (I:C)- and IFN-γ-activated RAFLS and SScHDF (panels A–D). Of note, transfection of miR-30a-3p mimic did not modulate IL-6 secretion by RAFLS and SScHDF activated with Poly (I:C) or IFN-γ (figure 4E–F), which is another major cytokine involved in B cells proliferation [23]. This indicates that miR-30a-3p does not interact with transcripts encoding factors involved in cytokine expression or inflammatory responses, but rather specifically interferes with BAFF mRNA in these cells, hence strengthening a potential role for BAFF in this process. Similar results were obtained upon transfection of miR-30e-3p and miR-30d-3p (data not shown).

These data demonstrate that miR-30a-3p is implicated in the negative regulation of BAFF synthesis in Poly (I:C)- and IFN-γ-activated FLS and HDF.

### miR-30a-3p specifically represses BAFF-dependent B cells survival

**Figure 5:**
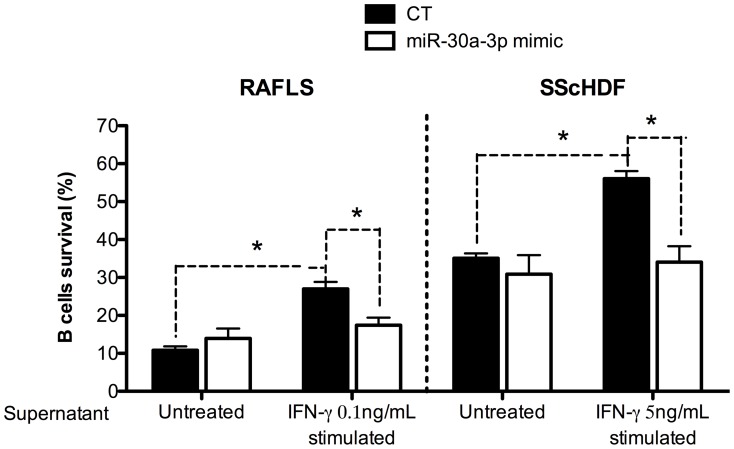
miR-30a-3p expression in RAFLS and SScHDF regulates BAFF-dependent B cells survival in vitro. RAFLS (n = 4) (left) and SScHDF (n = 4) (right) were transfected with miR-30a-3p mimic (20 pM/sample) or with an AllStars negative control (CT). After 24 h, cells were activated with IFN-γ (0.1 or 5 ng/mL depending on the cell type) or medium for 72 h. Then, supernatants were harvested and cultured with purified blood B cells isolated from healthy subjects. B cells viability was determined by FACS analysis; vital B cells were brightly positive when stained with DiOC6 and excluded PI. Data are expressed as the mean of triplicate samples ± SEM. *p<0.05.

Next, we checked the physiological relevance of BAFF regulation by miR-30a-3p. To this end, we measured the faculty of stimulated RAFLS and SScHDF to promote B cells survival following transfection of miR-30a-3p mimic. RAFLS and SScHDF were transfected with miR-30a-3p mimic or with the AllStars negative control siRNA (CT) for 24 h and then activated with IFN-γ for 72 h. The supernatants (conditioned medium) were harvested and added in the culture medium of B cells. After 3 days, survival of CD19-gated B cells was assessed by FACS analysis. As shown in figure 5, addition of supernatant from IFN-γ-stimulated fibroblasts significantly (p<0.05) increases the proportion of viable B cells, which shifts from 10 to 26% in the case of RAFLS supernatant and from 35 to 56% in the case of SScHDF. Importantly, increasing miRNA activity upon transfection of miR-30a-3p mimic in activated fibroblasts lowers B cells viability (15% and 35%, respectively. p<0.05). Additionally, we analyzed the supernatant of Poly (I:C)-activated RAFLS and SScHDF but we could not detect any difference in B cells survival (data not shown). Indeed, Poly (I:C), unlike IFN-γ, is also a potent inducer of IL-6 release by RAFLS and SScHDF (figure 4 E–F) which could regulate B cells survival. Similar results were obtained upon transfection of miR-30e-3p and miR-30d-3p (data not shown).

This experiment reveals that the modulation of miR-30a-3p activity induces important physiological changes with potentially relevant immune repercussions within the frame of autoimmune diseases.

### miR-30a-3p is a basal repressors of BAFF expression in non-inflammatory fibroblasts (NFLS and NHDF)

**Figure 6:**
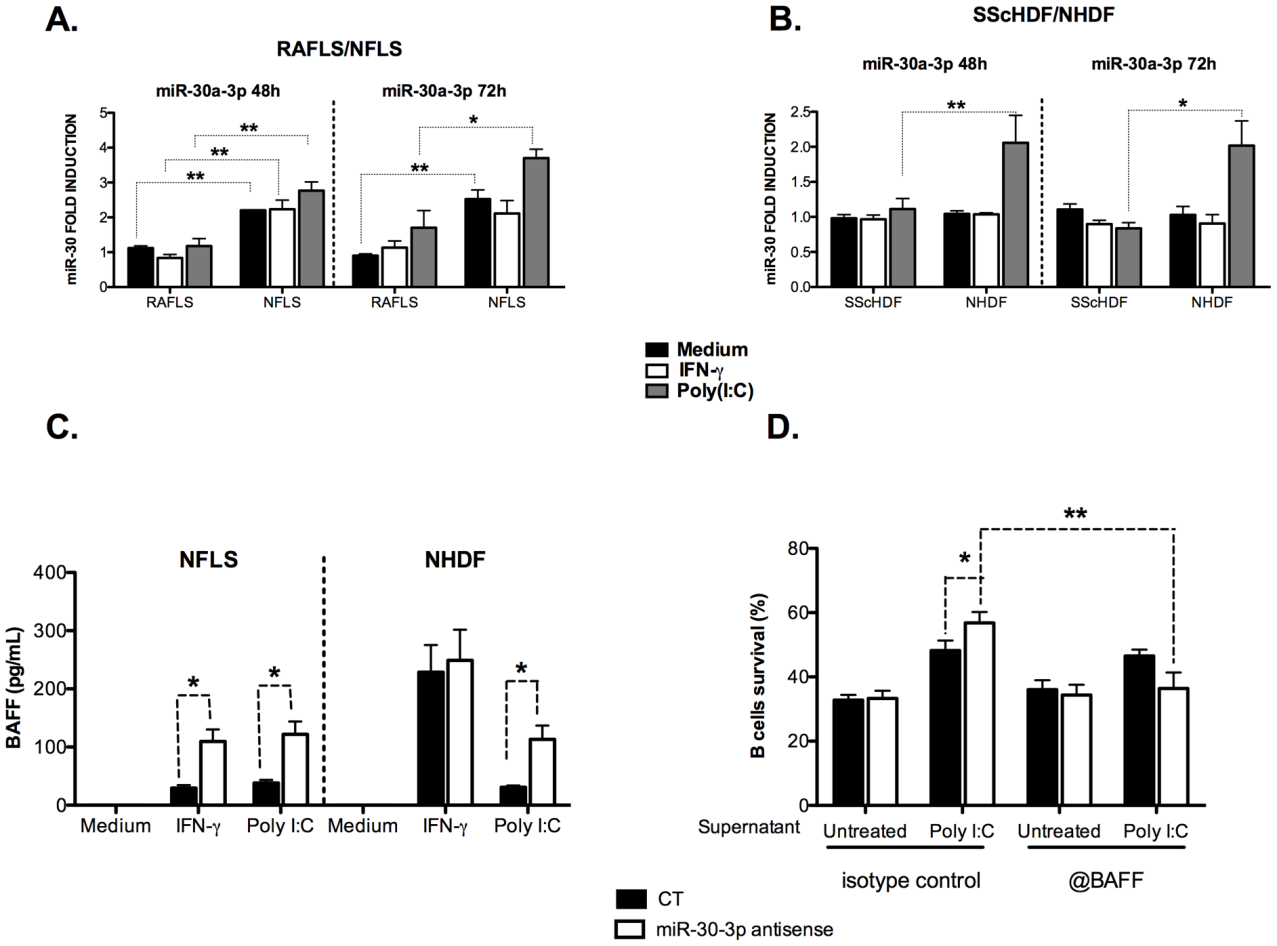
MiR-30a-3p represses BAFF secretion by healthy FLS and HDF. A, B. miR-30a-3p expression was determined by RT-qPCR in NFLS (n = 4)/RAFLS (n = 4) (A) and NHDF (n = 3)/SScHDF (n = 4) (B) stimulated with Poly(I:C) (10 µg/mL), IFN-γ (0.1 or 5 ng/mL) or medium for 48 h and 72 h. Results were normalized to U6snRNA and expressed as fold change compared with samples from RAFLS (A) or SScHDF (B) incubated with medium. C. NFLS (n = 3) and NHDF (n = 3) were transfected with miR-30-3p antisense oligonucleotides (20 pM/sample) or with an AllStars negative control (CT). After 24 h, cells were activated with Poly(I:C) (10 µg/mL), IFN-γ (0.1 or 5 ng/mL) or medium for 72 h. BAFF release was determined by ELISA in culture supernatants. D. NHDF (n = 3) transfected with miR-30-3p antisense, were stimulated with poly (I:C). The supernatant was then treated with control IgG or with anti-BAFF antibodies and added to purified B cells. B cells survival was next evaluated as in panel Figure 5. *p<0.05; **p<0.01.

Finally, we considered the possibility that miR-30a-3p family members could represent an essential mechanism to maintain BAFF expression at a very low level necessary in healthy conditions. Indeed, excessive BAFF secretion leading to increased B cells activation constitutes a major trigger promoting auto-immunity. A first insight into this model is provided by our initial observation of augmented BAFF secretion in the supernatant of RAFLS compared to NFLS in response to Poly(I:C) and IFN-γ (figure 1B). Therefore, we compared the expression of miR-30a-3p between healthy donor and patients cells in response to Poly(I:C) and IFN-γ for 48 h and 72 h. Interestingly, we showed that control NFLS as well as Poly(I:C)- and IFN-γ-stimulated cells expressed higher levels of miR-30a-3p compared to RAFLS under the same conditions (figure 6A). Moreover, NHDF which did not release BAFF in response to Poly(I:C) (figure 1D), also expressed higher levels of miR-30a-3p in the same conditions (figure 6B). These observations strongly suggest that miR-30a-3p could be a basal repressor of BAFF expression in these cells. Similar results were obtained upon transfection of miR-30e-3p (data not shown).

To test this hypothesis, we next lowered the activity of miR-30a-3p by transfecting specific antagonists (antisense 2′O methylated oligoribonucleotides) or negative controls (CT) in NFLS and NHDF. 24 h after transfection, cells were stimulated with Poly(I:C) or IFN-γ for 72 h. As illustrated in figure 6C, treatment with Poly(I:C) significantly induced higher levels of BAFF release by activated NHDF transfected with antisense oligonucleotides targeting miR-30a-3p compared to activated NHDF transfected with control (CT) (113,15±23,75 *vs* 30,75±3,25 pg/ml). A similar increase in BAFF secretion was obtained in NFLS transfected with antisense oligonucleotides targeting miR-30a-3p stimulated with Poly(I:C) (122±22 vs 38,2±5,5 pg/ml) and IFN-γ (109,45±20,85 vs 29,65±4,85 pg/ml). This experiment suggests that miR-30a-3p could represent an essential mechanism to maintain BAFF expression at a very low level necessary in healthy conditions. Moreover, we checked whether BAFF transcripts modulation upon miR-30a-3p inhibition also positively impacts on B cells survival. For this, we added anti-BAFF antibodies to poly (I:C)-stimulated NHDF treated with miR-30-3p antagomiRs. As seen in figure 6D, addition of anti-BAFF antibodies significantly (p<0.01 **) eradicates miR-30a-dependant-B cells survival improvement, thereby demonstrating that miR-30a-3p specifically modulates BAFF expression.

## Discussion

The role of B cells in autoimmunity has undergone a major renaissance after the demonstration of the efficacy of B cells depletion in RA [24]. BAFF plays a pivotal role in B cells activation in autoimmune diseases and is secreted by resident cells of target organs such as fibroblast-like synoviocytes [22], [25], [26]. On the other hand, the pivotal role of innate immunity in the initiation of autoimmune diseases is now well established, which prompted our present investigation on the role of innate immune receptors ligands and interferon-γ on BAFF secretion. We performed our analysis in fibroblasts isolated either from the skin (HDF) of healthy donors or from patients suffering from SSc or from the synovium (FLS) of RA patients or controls. Our results clearly show that, while Poly (I:C) stimulation induces high levels of BAFF transcription, LPS or BLP (respectively ligands of TLR4 and TLR2) remain poor activators of BAFF expression and secretion. Likewise, Poly (I:C) as well as LPS or BLP did not induce the synthesis of APRIL (a proliferation inducing ligand), which regulates also lymphocyte survival and activation (data not shown). This finding is consistent with results from our group which proposed that TLR2, 4 and 9 ligands failed to induce BAFF mRNA and protein in rheumatoid FLS [22]. Likewise, stimulation of salivary gland epithelial cells (SGEC) obtained from patients with primary Sjögren's syndrome (pSS) or bronchial epithelial cells with TLR2, 7 and 9 ligands does not induce BAFF transcription and secretion [27], [28]. Therefore, a first important result of this study is our description of specific Poly (I:C)-dependent BAFF transcriptional induction and subsequent secretion by SScHDF. This observation provides a conceptual framework whereby pathogens, such as herpesviruses which are capable of triggering a TLR3-dependent response [29] and have been associated to many autoimmune diseases [30], can initiate BAFF secretion and ignite a vicious circle leading to pathogenic auto-antibodies production. In line with this hypothesis, Ittah et al. suggested that PKR is the major mediator of BAFF expression and secretion after dsRNA virus infection or Poly (I:C) stimulation by salivary epithelial cells of pSS [31].

Next, we investigated the mechanisms involved in the regulation of BAFF production by FLS and HDF. miRNAs, which are considered as efficient fine tuners of immune responses because they usually modulate gene expression by a factor 1.2 to 4 [32], exhibit abnormal expression associated with inflammatory disorders such as RA [33], [34], SLE [35], [36] and SSc [37], [38]. In an initial attempt to identify miRNAs involved in the control of BAFF expression, we performed RT-qPCR analysis of Poly (I:C)- and IFN-γ-activated RAFLS and SScHDF to quantify the expression of 9 miRNAs that were predicted to target BAFF transcripts. Among these candidates, miR-30a-3p was selected for further analysis because its expression was down regulated in Poly (I:C)- and IFN-γ-activated RAFLS and SScHDF; such inverse correlation with the expression of BAFF transcripts in the same cells and the same conditions indicated that miR-30-3p family members might have a role in the regulation of BAFF expression. We next demonstrated by several complementary approaches that miR-30a-3p actually bind specifically to the 3′UTR of BAFF transcripts and modulate cytokine expression. Importantly, additional results (data not shown) indicate that additional members (and closely related) of the miR-30a-3p family (miR30-d-3p and -e-3p) also regulate BAFF expression in RAFLS and SScHDF stimulated with Poly (I:C) or IFN-γ. Therefore, our study additionally suggests a novel mechanism for the regulation of BAFF expression at the posttranslational level in response to inflammatory stimuli but the transcriptional regulation of BAFF expression must also be considered. Usually, miRNAs can function together with RNA-binding proteins to regulate mRNA expression through the AU-rich elements (AREs) that are found in numerous cytokine-encoding mRNAs. For example, TNF-α and IL-10 mRNAs both contain long AREs that are targeted by the RNA-binding protein tristetraprolin (TTP) [39]. Our group previously showed that blocking miR-346 decreases TTP expression and re-established mature TNF-α intracellular expression in LPS-activated RAFLS [40], [41]. Although evidence for the direct targeting of cytokine mRNAs by miRNAs is limited [39], we demonstrated in this study that miR 30a-3p directly regulates BAFF mRNA.

Importantly, we also analyzed in our study the functional outcome of miRNA-dependent BAFF regulation. We demonstrated that IFN-γ stimulation of fibroblasts favors an extracellular milieu that promotes B cells survival. Importantly, our experiments (illustrated in Figure 5) demonstrated that specifically altering BAFF secretion (with miR-30a mimic or upon anti-BAFF antibodies addition following antagomiRs transfection) reduces the B cells survival capacity of supernatant from IFN-γ stimulated RAFLS or SScHDF back to normal (as observed in control cells). This indicates that BAFF (and not IL-6) is the major B cells survival factor expressed by these fibrocytes upon IFN-γ stimulation. Similar to our findings, Ohata et al reported that FLS treated with IFN-γ and/or TNF-α had a greater capacity to support B cells survival than did untreated FLS [26]. B cells survival could be inhibited by BAFF-R:Fc, indicating that BAFF/BAFF-R interactions were involved in B cells survival [26]. Altogether, this work provides a mechanistic explanation to the control of BAFF transcripts expression and demonstrates that cytokine secretion by resident cells of target organs of autoimmune diseases can be negatively regulated at the post-transcriptional level by miRNAs. A tentative model describing these interactions is depicted in Figure 2S. The understanding of these complex pathways has important implications for the development of future therapeutic applications. Indeed, the success of Belimumab in the treatment of patients with RA and ongoing clinical trials in SSc (NCT01670565) suggest that therapeutic targeting of BAFF could be of interest [42]. Our present study suggests that miR-30a-3p (and others family members) mimic could be used to target BAFF mRNA in autoimmune diseases. Recently, patients chronically infected with hepatitis C virus (HCV) treated with locked nucleic acid (modified antisense oligonucleotides) against miR-122 showed a prolonged dose-dependent reductions in HCV RNA levels without evidence of viral resistance [43], which suggests that miRNA modulation in patients could become a new therapeutic option in the future.

## Supporting Information

Figure S1 miRNAs expression in RAFLS and SScHDF. MiR-144*, miR-30d-3p, miR-452, miR-340, miR-202, miR-500, miR-626, miR-330-3p and miR-302c* expression was determined by RT-qPCR in RAFLS (n = 3) and SScHDF (n = 3) stimulated with Poly (I:C) (10 µg/mL) or IFN-γ (0.1 or 5 ng/mL) for 72 h. Results were normalized to U6snRNA and expressed as fold change compared with samples from RAFLS or SScHDF incubated with medium.
(TIFF)

Figure S2 **Model describing the role of miR-30a-3p in BAFF secretion by FLS (A) and HDF (B) from RA or SSc patients and healthy subjects.**
(TIFF)
