# Supplementary figures and images for: MiR-30a-3p Negatively Regulates BAFF Synthesis in Systemic Sclerosis and Rheumatoid Arthritis Fibroblasts
Source: PLoS One. 2014 Oct 31;9(10):e111266. doi: 10.1371/journal.pone.0111266 (PMC4216016; doi:10.1371/journal.pone.0111266)

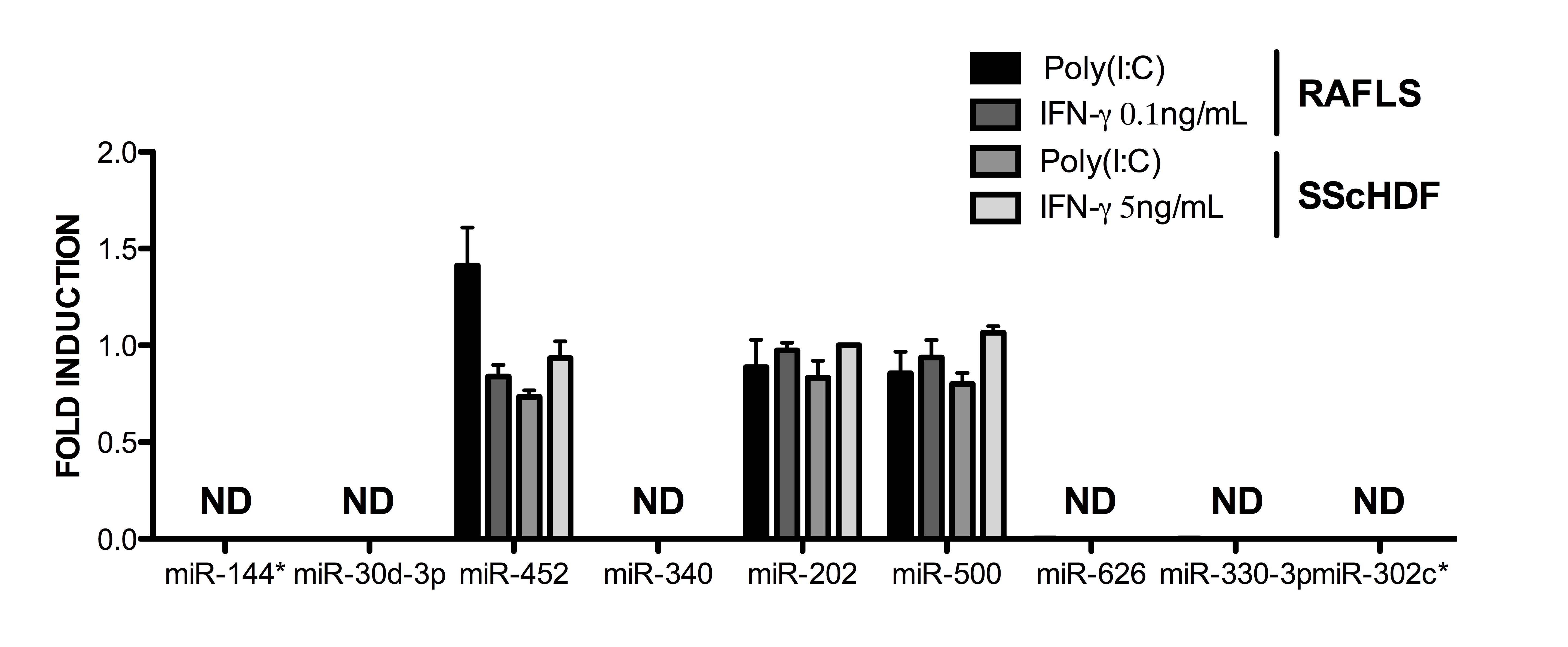

Supplement: Figure S1 — miRNAs expression in RAFLS and SScHDF. MiR-144*, miR-30d-3p, miR-452, miR-340, miR-202, miR-500, miR-626, miR-330-3p and miR-302c* expression was determined by RT-qPCR in RAFLS (n = 3) and SScHDF (n = 3) stimulated with Poly (I:C) (10 µg/mL) or IFN-γ (0.1 or 5 ng/mL) for 72 h. Results were normalized to U6snRNA and expressed as fold change compared with samples from RAFLS or SScHDF incubated with medium. (TIFF) [file pone.0111266.s001.tiff]

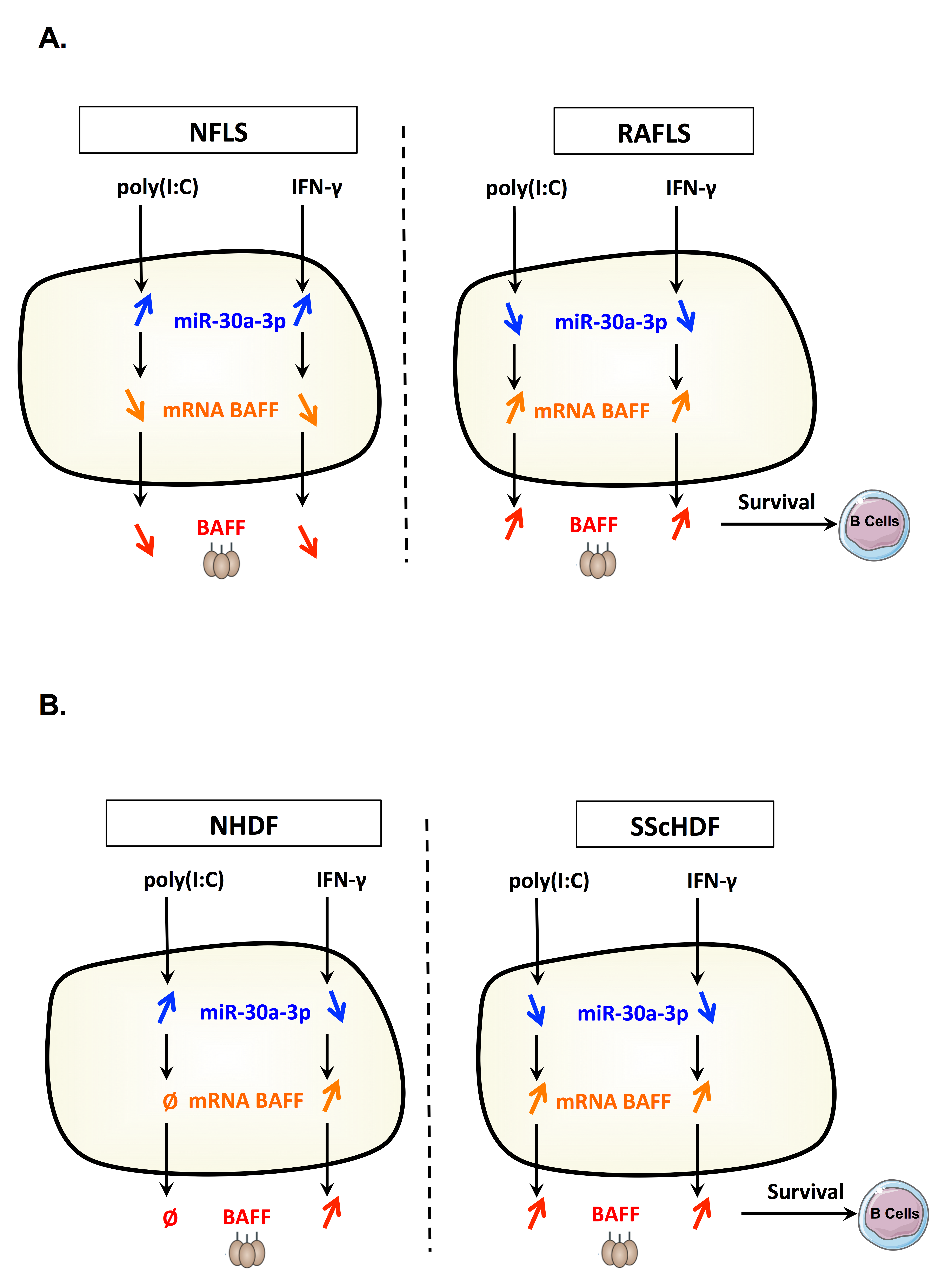

Supplement: Figure S2 — Model describing the role of miR-30a-3p in BAFF secretion by FLS (A) and HDF (B) from RA or SSc patients and healthy subjects. (TIFF) [file pone.0111266.s002.tiff]
